# Intraoperative radiation therapy with the photon radiosurgery system in locally advanced and recurrent rectal cancer: retrospective review of the Cleveland clinic experience

**DOI:** 10.1186/1748-717X-7-110

**Published:** 2012-07-20

**Authors:** Susan Guo, Chandana A Reddy, Matthew Kolar, Neil Woody, Arul Mahadevan, F Christopher Deibel, David W Dietz, Feza H Remzi, John H Suh

**Affiliations:** 1 Department of Radiation Oncology, Cleveland Clinic, T28 9500 Euclid Ave., Cleveland, OH, 44195, USA; 2 Department of Colorectal Surgery, Cleveland Clinic, Cleveland, OH, USA; 3 Department of Radiation Oncology, Lahey Clinic, Peabody, MA, USA

**Keywords:** Colorectal cancer, Intraoperative radiation therapy, Local disease relapse, Salvage, Radiation tolerance

## Abstract

**Background:**

Patients with locally advanced or recurrent rectal cancer often require multimodality treatment. Intraoperative radiation therapy (IORT) is a focal approach which aims to improve local control.

**Methods:**

We retrospectively reviewed 42 patients treated with IORT following definitive resection of a locally advanced or recurrent rectal cancer from 2000–2009. All patients were treated with the Intrabeam® Photon Radiosurgery System (PRS). A dose of 5 Gy was prescribed to a depth of 1 cm (surface dose range: 13.4-23.1, median: 14.4 Gy). Median survival times were calculated using Kaplan-Meier analysis.

**Results:**

Of 42 patients, 32 had recurrent disease (76%) while 10 had locally advanced disease (24%). Eighteen patients (43%) had tumors fixed to the sidewall. Margins were positive in 19 patients (45%). Median follow-up after IORT was 22 months (range 0.2-101). Median survival time after IORT was 34 months. The 3-year overall survival rate was 49% (43% for recurrent and 65% for locally advanced patients). Local recurrence was evaluable in 34 patients, of whom 32% failed. The 1-year local recurrence rate was 16%. Distant metastasis was evaluable in 30 patients, of whom 60% failed. The 1-year distant metastasis rate was 32%. No intraoperative complications were attributed to IORT. Median duration of IORT was 35 minutes (range: 14–39). Median discharge time after surgery was 7 days (range: 2–59). Hydronephrosis after IORT occurred in 10 patients (24%), 7 of whom had documented concomitant disease recurrence.

**Conclusions:**

The Intrabeam® PRS appears to be a safe technique for delivering IORT in rectal cancer patients. IORT with PRS marginally increased operative time, and did not appear to prolong hospitalization. Our rates of long-term toxicity, local recurrence, and survival rates compare favorably with published reports of IORT delivery with other methods.

## Introduction

Treatment for locally advanced rectal cancer consists of multiple modalities including surgery, radiation, and chemotherapy. Many locally advanced lesions can be rendered resectable with preoperative chemotherapy and radiation, and modern recurrence rates range from 6-10% after neoadjuvant therapy [1-4]. Local recurrence leads to significant morbidities including pelvic pain, bleeding, bowel obstruction, and poor quality of life. Intraoperative radiation therapy (IORT) has been used as part of a multimodality approach in an attempt to improve local control in this patient population.

Similarly, recurrent rectal cancer can be extremely debilitating. In the setting of recurrence, the likelihood of a margin negative resection is low [5]. The historical survival rates following surgical resection alone have been poor, with one series reporting a zero percent 5-year overall survival [6]. Furthermore, a majority of these patients have been previously treated with external beam radiation therapy (EBRT), which may preclude the delivery of additional pelvic radiation. IORT has been used in an attempt to increase local control in these patients. Using a single institutional database of patients treated with IORT for rectal cancer, we retrospectively reviewed our experience with the photon radiosurgery system (PRS) in the delivery of IORT for locally advanced and recurrent rectal cancer patients.

## Methods and materials

From an IRB-approved registry, we retrospectively reviewed patients treated with IORT following definitive resection of locally advanced or recurrent rectal cancer at our institution. All patients were treated with the Intrabeam® Photon Radiosurgery System (Carl Zeiss, Oberkochen, Germany).

The Intrabeam device provides a point source of low energy x-rays (50 kV maximum) at the tip of a 3.2 mm diameter tube that is placed at the center of a spherical tumor bed applicator. The diameter of the spherical applicators ranges from 1.5-5 cm in 0.5 cm increments. Spherical applicators ranging from 2–5 cm were used for the patients in our review.

During the operative procedure, calibration and quality checks are performed by the medical physicist. In the case of primary rectal cancers, surgery is performed following the principles of total mesorectal excision. The hypogastric nerves are identified at the level of the sacral promontory and are preserved. Likewise, the endopelvic fascia covering the pelvic sidewall is not violated so as to maintain protection of the autonomic plexus bilaterally. In cases where the rectal cancer is not located anteriorly, Denonvillier's fascia is preserved (not included with the resection specimen) so as to minimize the risk of injury to the nervi erigentes. These technical details allow for very low rates of sexual and urinary dysfunction after total mesorectal excision, even with the additional effects of external beam pelvic radiation. Resections for recurrent rectal cancer are carried out in the anatomic plane that will provide the best chance for a tumor-free radial margin. These operations often involve the enbloc resection of adjacent viscera (vagina, uterus, bladder, sacrum, etc.) to achieve R0 status. In cases where a restorative procedure is being performed (colorectal or coloanal anastomosis), IORT is delivered prior to the creation of the anastomosis when the pelvis is largely empty. Areas of concern for a close or involved radial margin are identified and marked by the surgeon. The largest possible applicator that can be placed into the tumor bed is selected and locked into the x-ray unit. The spherical applicator is placed in the tumor bed by surgeon and radiation oncologist. Thin sheets of lead, covered in sterile plastic drape, are then applied to shield normal structures such as the small bowel and ureters in or near the radiation field. The operating room personnel wear leaded aprons and stand behind a shields screen while IORT is delivered.

In all patients, a dose of 5 Gy was prescribed to a depth of 1 cm (surface dose – based on applicator diameter- range: 13.4-23.1 Gy, median: 14.4 Gy). This dose was selected based on our institutional experience in using the same dose to treat microscopic and subclinical disease. This was also the dose used in the TARGIT trial, whose primary goal was to eradicate microscopic and subclinical disease in breast cancer after lumpectomy [7]. Resection status in our cohort were as follows: R0 in 22 patients (52.4%), R1 in 19 patients (45.2%), and R2 in 1 patient (2.4%). In the sole patient with R2 resection, the residual tumor was larger than the surface of the applicator (length 5 cm, width 3 cm, depth 3 mm); after discussion with the colorectal surgeons, it was felt that IORT would be beneficial to palliate the bulk of disease. Therefore, the dose was not altered to account for the R2 resection. Prescribing the dose to 1 cm depth ensured full dose at this depth, which was felt to be at risk for microscopic disease. The surface dose was higher, commensurate with the applicator diameter.

Ureteral stents were placed at the time of surgery for identification of ureters in 76% of patients. Median survival times, defined as survival after the IORT procedure, were calculated using Kaplan-Meier analysis. In patients with recurrent disease, IORT was delivered at the time of salvage surgical resection. Local recurrence was defined as failure within the pelvis, and distant metastasis was defined as metastatic disease outside the pelvis.

## Results

We analyzed 42 patients treated between 2000 and 2009 (Table 1). The median age was 56 (range: 24–80 years). Thirty-seven patients (88%) were previously treated with pelvic external beam radiation therapy. The median dose was 5040 cGy (range: 2700–6300 cGy). Dates of pre-operative radiotherapy were available in 26 patients. In these 26 patients, the median time from completion of prior radiation to time of surgery and IORT was 3 months (range 1–74 months). Eleven additional patients from outside facilities had external beam radiation therapy prior to IORT but did not have a completion date available. Thirty-six patients (85.7%) underwent chemotherapy prior to IORT. Of patients who received chemotherapy, the regimen was 5-FU-based in 31 patients (86.1%) and unknown in 5 patients (13.9%).

**Table 1:** Patient Characteristics

	**n**	**%**
**Gender**		
F	16	38.1
M	26	61.9
**Initial Stage at Diagnosis**		
I	9	21.4
II	14	33.3
III	11	26.2
IV	3	7.1
Unknown	5	11.9
**Pathology**		
Adenocarcinoma	40	95.2
Carcinoma, poorly differentiated	1	2.4
Malignant carcinoid	1	2.4
**Recurrent vs. Advanced**		
Locally advanced	10	23.8
Recurrent	32	76.2
**Fixation**		
N	24	57.1
Y	18	42.9
**Pelvic radiation before IORT**		
N	5	11.9
Y	37	88.1
**Margin Status at IORT**		
Negative	21	50.0
Positive	19	45.2
Unknown	2	4.8
**Resection Status**		
R0	22	52.4
R1	19	45.2
R2	1	2.4
**Chemotherapy before IORT**		
N	6	14.3
Y	36	85.7
**Chemotherapy Agents**		
5-FU based	31	86.1
Unknown	5	13.9

The median applicator size used was 5 cm (range: 2–5 cm). Median tumor size at IORT was 2.8 cm (range: 0–10 cm) based on operative report. Thirty-two patients had recurrent disease (76%) while 10 (24%) had locally advanced disease at presentation. For the purpose of the analysis, locally advanced disease was defined as tumors that were assessed to be unresectable without leaving microscopic or gross residual disease at the resection site. Fixed disease was defined by the operative report, and refers to tumors that were adherent to adjacent structures. Forty patients (95%) had adenocarcinoma histologies, and 18 patients (43%) had tumors fixed to the sidewall. Median follow-up after IORT was 22 months (range 0.2-101).

**Figure 1:**
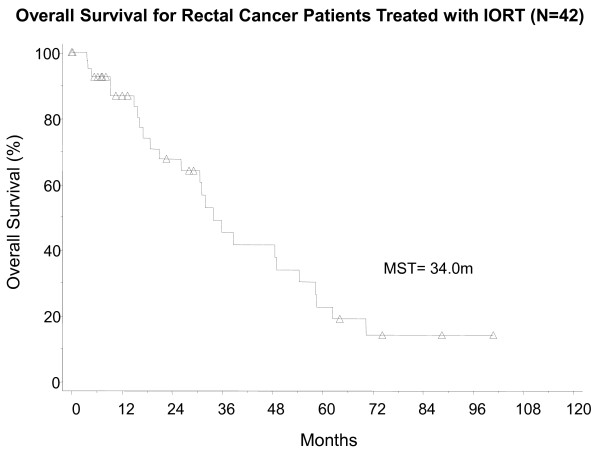
Overall Survival for All Rectal Cancer Patients Treated with IORT.

**Figure 2:**
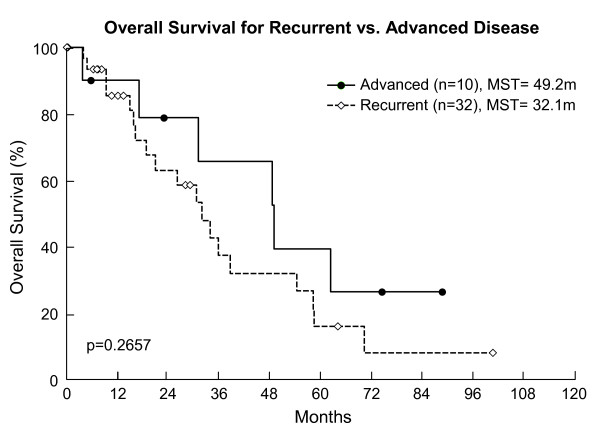
Overall Survival for Recurrent vs. Locally Advanced Disease Treated with IORT.

**Figure 3:**
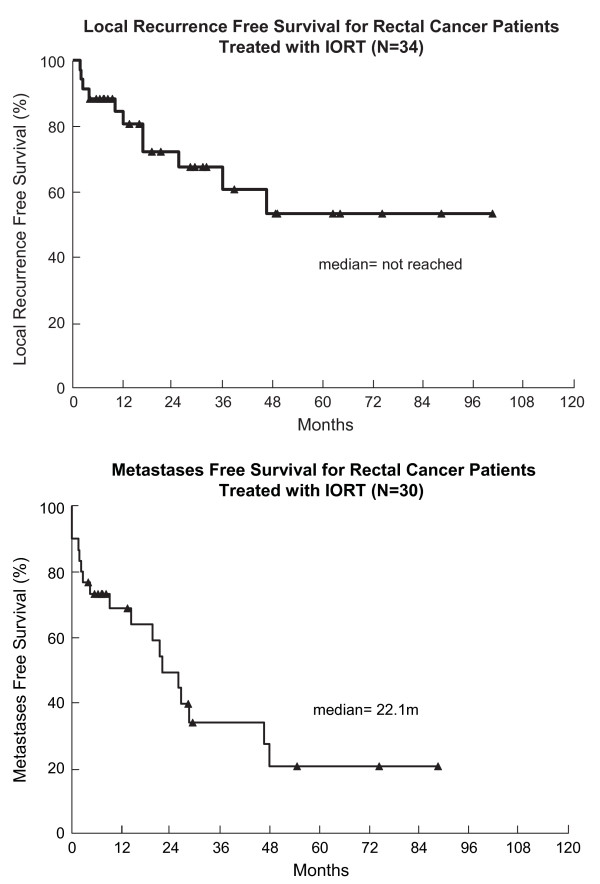
a) Local Recurrence Free Survival for All Rectal Cancer Patients Treated with IORT. b) Distant Metastasis Free Survival for All Rectal Cancer Patients Treated with IORT.

Median survival time after IORT was 34 months (Figure 1). The 3-year overall survival rate was 49%(95% CI 31-67%). In our subsets, the 3-year overall survival rate was 43% for patients with recurrent disease (95% CI 22-64%) and 65% (95% CI 33–97.5%) for patients with locally advanced disease (Figure 2). Local recurrence was defined as recurrence in the pelvis, and was evaluable in 34 patients. The remaining 8 patients did not have adequate follow-up information to address local disease status. Thirty-two percent of the evaluable patients failed locally. The 1-year local recurrence rate was 16% (95% CI 3-28%)(Figure 3a). The median time to local recurrence was 36 months for the subset of patients with recurrent disease. The median time to local recurrence was not reached in the subset of patients with locally advanced disease, due to small patient numbers, none of whom had local recurrence. Distant metastasis was evaluable in 30 patients, of whom 60% failed. The remaining 12 patients did not have adequate follow-up information to ascertain the status of distant failure. The 1-year distant metastasis rate was 32% (95% CI 14-49%)(Figure 3b). No intraoperative complications were attributed to IORT.

Median duration of IORT was 35 minutes (range: 14–39). Median discharge time after surgery was 7 days (range: 2–59). IORT did not appear to prolong hospitalization. Hydronephrosis after IORT was seen in 10 patients (24%).

## Discussion

**Figure 4:**
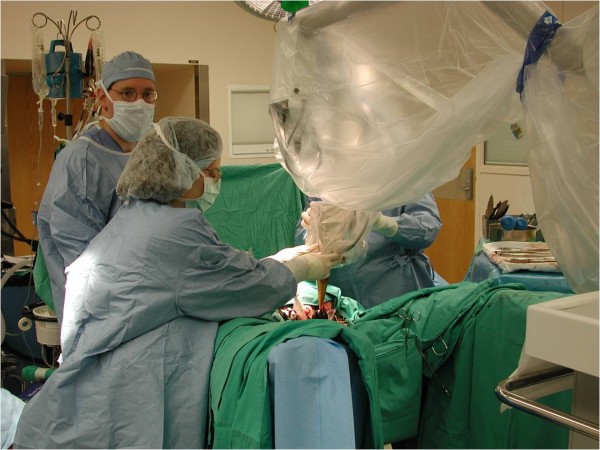
Positioning the Intrabeam® Photon Radiosurgery System in the Tumor Bed.

Our series is the first manuscript reporting results of treating locally advanced or recurrent rectal cancer with the Intrabeam® Photon Radiosurgery System as a component of multimodality therapy. Our results are consistent with those of other IORT series for locally advanced and recurrent rectal cancer (Figure 4). In the US, the Mayo Clinic and the Massachusetts General Hospital have published extensively on their experience with IORT with electron beams via linear accelerators, with energies ranging from 6 to 20 MeV. Haddock et al. reported their series of 607 patients with recurrent colorectal cancer who received IORT from 1981 to 2008 at the Mayo Clinic [8]. Their series showed a median overall survival of 36 months. Five-year survival was 30%. Three-year local control and distant metastases rates were also comparable to our series. Similarly, the Massachusetts General Hospital reported outcomes of 49 patients with locally recurrent rectal carcinomas without metastases treated with IORT from 1978 to 1997 [9]. Five-year survival was 27%, 5-year local control was 35%, and the overall rate of distant metastases was 67%.

European IORT series with electron beams have also reported similar rates of local control and survival. A prospective study from Norway showed that IORT in 59 patients yielded a 5-year local control rate of 30% in R0 patients and 50% in R1 patients [10]. Five- year survival was 30% and highly depended on the volume of residual disease (5-year survival 60% R0, 25% R1, and 0% R2). A large series from the Netherlands reported results of 147 patients treated with IORT [11]. Five-year local control rate was 54% and median survival was 28 months. Radical resection was significantly correlated with improved local control and overall survival. One series from Spain reporting on IORT in 27 patients yielded a 2-year local control rate of 26%, distant metastasis rate of 41% and 5-year survival of 12% [12]. The authors attributed these higher rates of local failure to the significant percentage of incomplete resections in their cohort, 66% of all patients. These major series of IORT for recurrent colorectal cancer are summarized in Table 2[13].

**Table 2:** Comparison of the Cleveland Clinic’s Data with Major Series of Patients with Recurrent Disease treated with IORT

**Series**	**# Pts**	**Local Control**	**Distant Metastases**	**Survival**
MayoClinic (8)	607	3-year: 65%	3-year: 49%	MS: 36 mo
				5-year: 30%
MGH(9)	49	5-year: 35%	Overall: 67%	5-year: 27%
Norway (10)	59	5-year:	Not Reported	5-year: 30%
		R0: 70%		
		R1: 50%		
Netherlands (11)	147	5-year: 54%	Not Reported	MS: 28 mo
				5-year: 32%
Spain (12)	27	2-year: 26%	41%	5-year: 12%
France (13)	73	3-year: 31%	Not Reported	3-year: 31%
Cleveland Clinic	32	3-year: 56%	3-year: 64%	MS: 32 mo
				5-year: 16%

Although there were only 10 patients with locally advanced disease in our series, our outcomes were comparable to other IORT series of this patient population. We observed 3-year local control of 100%, 3-year distant metastasis rate of 71%, and 5-year survival of 39%. The median survival was 49 months. Mathis et al. published the Mayo experience with 146 locally advanced rectal cancer patients treated with IORT and observed a median survival of 44 months [14]. They also observed 3-year local control of 90%, 3-year distant metastasis rate of 43%, and 5-year survival of 52%. Willet et al. retrospectively reviewed the series at Massachusetts General Hospital of 65 patients who underwent resection with the intention of using IORT if areas at high risk for local recurrence were apparent at surgery, 42 of whom ultimately received IORT [15]. Local control of 77% was seen at 5 years; distant metastases and overall survival rates were not reported.

Large European series have reported similar good outcomes in this patient population. Diaz-Gonzalez et al. reported their experience in Madrid with 115 T3-4 or N + patients treated with preoperative chemoradiation, surgery, and IORT [16]. Their 3-year local control was 34% and distant metastasis rate was 21%. The 3-year median survival was 74%. Krempien et al. reported long-term results from the University of Heidelberg in 210 locally advanced rectal cancer patients treated with chemoradiation, TME, and IORT [17]. Their 5-year local control rate was 93%. The 5-year survival was 69%. In these series, resection status was prognostic for improved disease-free survival and local control, respectively. Comparison of these major series with our results is summarized in Table 3.

**Table 3:** Comparison of the Cleveland Clinic’s Data with Major Series of Patients with Locally Advanced Disease treated with IORT

**Series**	**# Pts**	**Local Control**	**Distant Metastases**	**Survival**
MayoClinic(14)	146	3-year: 90%	3-year: 43%	MS: 44 mo
				5-year: 52%
MGH(15)	42	5-year: 77%	Not Reported	Not Reported
Madrid (16)	115	3-year: 94%	3-year: 21%	MS: Not Reached
				3-year: 74%
Heidelberg (17)	210	5-year: 93%	Not Reported	5-year: 69%
Cleveland Clinic	10	3-year: 100%	3-year: 71%	MS: 49 mo
				5-year: 39%

In the Mayo series, the largest series of patients with recurrent disease treated with IORT, toxicity grade 3 or higher partially attributable to IORT was seen in 66 patients, mostly consisting of wound infection, abscess, or fistula (cumulatively 7%), ureteral obstruction (3%), and neuropathy (3%). Any grade neuropathy was observed in 15% of patients and was more commonly seen with IORT doses exceeding 12.5 Gy [8]. In our series, of the 10 patients who were found to have hydronephrosis after IORT, 7 had documented concomitant disease recurrence, and the etiology of hydronephrosis is likely multifactorial. Peripheral neuropathy was not identified as a toxicity in our series. However, our toxicities rates may be limited due to our relatively short follow-up time. A subset of our patients were referred to our center for IORT only and subsequently discharged home; these patients were not included in the analysis of toxicities due to lack of follow-up information. Reporting of toxicities was also limited because of the retrospective nature of this analysis. Prospective documentation of follow-up visits as well as thorough assessment of expected toxicities are necessary to detail toxicities of this emerging treatment modality. Also due to the retrospective nature of this analysis, we did not have follow-up information on 8 patients for local recurrence and 12 patients for distant metastases. Some of these patients were diagnosed with local recurrence or distant metastases at our institution but were subsequently lost to follow-up. Because our institution is a tertiary medical center, a number of patients traveled here solely for IORT and chose to be followed with their local physicians. However, the data that we report on the remaining evaluable patients still represents the largest pool of patients treated with the Intrabeam® PRS for colorectal cancer.

The only other report of using the Intrabeam® PRS for colorectal cancer in the literature is a series of twenty patients from Russia, presented as an abstract. Lyadov et al. reported their experience with 20 colorectal adenocarcinoma patients with T3-4 disease treated with this system after curative resection [18]. A dose of 14–17 Gy to the surface was prescribed. Five patients received minimally invasive surgery; in these patients, the IORT applicator was inserted through the incision that was used to remove resected material. Of these five patients, no local recurrences were seen. No early or delayed radiation toxicities were attributed to radiation, and no deaths occurred in the early postoperative period. Similar to our series, their mean duration of radiation exposure was 30 minutes. The authors proved the feasibility of this technique to be used with minimally invasive surgery, and although the length of follow-up is not given, they reported minimal complications attributable to IORT in the early postoperative period.

The Intrabeam® PRS has some limitations. The largest applicator, being 5 cm, restricts the area that can be treated. Intraoperative high dose-rate brachytherapy with ^192^Iridium and IORT with low energy electron beams can cover a much larger area. In certain clinical scenarios, IORT has been aborted because the target area could not be adequately covered with the largest applicator. Because our database was only able to query cases where IORT was delivered, we cannot provide an exact number of cases in which IORT was not possible. Other reasons for aborting IORT at our institution include unresectable disease and metastatic disease detected at the time of surgical exploration.

The dose-rate of the Intrabeam® PRS is lower than other modalities, which results in longer treatments. The median duration of IORT was 35 minutes in our series (range: 14–39). The percent depth dose of 50 kV X-rays is very steep with no build-up region. This creates inhomogeneity across the treatment volume, with the highest dose being at the surface (dose inhomogeneity index for this series – range: 2.68-4.62, median: 2.88).

## Conclusion

The use of Intrabeam® PRS appears to be a safe technique for delivering IORT in patients with locally advanced and recurrent rectal cancer. IORT with PRS marginally increased operative time, and did not appear to prolong hospitalization. Our rates of toxicity, local recurrence, and survival rates compare favorably with published reports of IORT delivery with electrons.

## Presented at European Society of Surgical Oncology (Bordeaux, FR, September 2010) and International Society of Intraoperative Radiation (Scottsdale, AZ, October 2010)

## Competing interests

The first author received travel funding from Carl Zeiss Meditec (Oberkochen, Germany), manufacturer of the Intrabeam® device, to present these research findings in abstract form. The study design, retrospective review, and manuscript creation were all conceived and performed without any outside input. The remaining authors disclose no other potential conflicts of interest.

## Authors’ contributions

JS, AM, and SG participated in the design of the study. SG and NW designed the database and acquired the clinical data. CR performed the statistical analysis of the data. MK and FCD drafted the physics portion of the manuscript. DD and FR helped draft the manuscript. All authors have reviewed the manuscript and approve of the final version to be published.
